# Get two for the price of one: *GmNF-YC4* factor mediates *GmEXP7*-induced root developmental changes and phosphorus starvation response in soybean

**DOI:** 10.1093/plphys/kiae554

**Published:** 2024-10-18

**Authors:** Héctor H Torres-Martínez

**Affiliations:** Plant Physiology, American Society of Plant Biologists; Department of Biology, Stanford University, Stanford, CA 94305, USA

Phosphorus (P) is an essential macronutrient for plant function and development. P is an essential component of cell membranes and nucleic acids and has critical roles in signaling pathways. Plants acquire this macronutrient through their roots and root hairs as inorganic phosphorus (Pi), (Bates and Lynch 1996). However, P availability is usually scarce because Pi is highly reactive, forming complexes with iron (Fe) and aluminum (Al), and it circulates between forms slowly in soils (Rhodes 2013).

In plants, Pi scarcity triggers alterations in root system architecture (RSA), which varies among species (Liu 2021).The typical response results in shallow root systems, accompanied by shortened and more branched roots with a higher density of longer root hairs (Liu 2021). At the molecular level, Pi deprivation starts the phosphorus starvation response (PSR), a conserved pathway involving the MYB transcription factors Pi STARVATION RESPONSE1 (PHR1) and PHR1-LIKE1 (PHL1) (Madison et al. 2023). PHR transcription factors regulate the expression of a number of genes associated with, but not limited to, phosphate transporters, phosphatases, and lipid modification proteins to boost Pi acquisition (Madison et al. 2023).

Nuclear Factor-Y (NF-Y) proteins are multimeric transcription factors formed by 3 subunits encoded by *NF-YA*, *NF-YB*, and *NF-YC* genes (Romier et al. 2003). In *Arabidopsis thaliana*, the NF-Y factor encoded by *HEME ACTIVATOR PROTEIN3b* (*HAP3b*) positively regulates root development by speeding up cell elongation in the root tip (Ballif et al. 2011). Genome-wide expression analysis identified 21 *NF-YA*, 32 *NF-YB*, and 15 *NF-YC* genes in the soybean (*Glycine max*) genome, and a survey based on their expression pattern showed that some of them are developmental and stress-response specific (Quach et al. 2015). Despite many reported functions of Nuclear Factor-Y (NF-Y) transcription factors in regulating root development and stress response, their role during PSR is unclear.

In this issue of *Plant Physiology*, Liu et al. (2024) characterized the functional role of a soybean NF-Y factor, specifically GmNF-YC4. The authors previously identified a role for it in the regulation of flowering time, but here they show that it also mediates RSA changes and PSR in soybean under Pi deprivation conditions.

Phenotypic analysis of the loss-of-function mutant *Gmnf-yc4* showed an altered RSA with longer primary roots compared with wild-type (WT) plants, an extended root hair zone, and a 39% increase in root hair length. Additionally, the *Gmnf-yc4* mutants had higher total biomass and P content than the WT when plants were grown in normal and low-P conditions.

The authors showed that multiple PSR-associated genes like the ethylene biosynthesis regulator *ETHYLENE OVERPRODUCTION PROTEIN 1* (*ETO1*) (Zhang et al. 2020), the Pi transporter *PHOSPHATE TRANSPORTER 9* (*GmPT9*) (Qin et al. 2012), and the cell wall–loosening protein *α-EXPANSIN 7* (*GmEXPA7*) (Lin et al. 2011), among others, were differentially expressed in the *Gmnf-yc4* background compared with WT, with a greater difference when plants were exposed to low-P conditions. Yeast 1-hybrid experiments showed that GmNF-YC4 interacts with the promoter of *GmEXPA7*, a previously reported root growth promoter, but not with the other PSR genes surveyed. Transient experiments on *Nicotiana benthamiana* leaves showed that *GmEXPA7pro:LUC* is repressed by GmNF-YC4. Further analysis showed that even though *GmEXPA7* is expressed across all tissues, it showed the highest expression in roots. Furthermore, *GmEXPA7* promoter activity was elevated in low-P conditions. When overexpressed, *GmEXPA7* increased root length, root volume, root biomass, and P content. Overall, these data suggest that the module GmNF-YC4-GmEXPA7 mediates the response to low-P conditions through the control of RSA.

Because GmNF-YC4 was shown to control the expression of additional PSR-related genes, the authors performed DNA affinity purification sequencing analysis to search for more targets. DNA affinity purification sequencing revealed a total of 5,092 potential target genes where GmNF-YC4 might be directly bind to their promoters. Gene ontology (GO) enrichment analysis showed that these putative target genes were enriched in pathways associated with PSR, like hormone receptor binding, transcription factor binding, regulation of response to stimulus, and regulation of phosphorus metabolic process. Moreover, GO analysis also showed that nitrogen (N) metabolism-related pathways were enriched. When *Gmnf-yc4* was analyzed for N content, it showed higher levels compared with WT, indicating that the GmNF-YC4 factor may act as an essential control point for N uptake and metabolism.

Overall, these findings increase our understanding of the complex mechanism of how plants respond to Pi deprivation. Liu et al. (2024) showed that GmNF-YC4 acts as a negative regulator of the low-P root-growth response by negatively regulating the expression of *GmEXPA7*, altering the RSA, and indirectly controlling the expression of other PSR genes (Fig.). However, questions remain about what other genes might be regulated by GmNF-YC4 and how it might indirectly regulate other genes in response to P starvation. Furthermore, it remains unclear how exactly Pi limitation modulates GmNF-YC4 expression and activity. Addressing these questions will help to connect the dots in how this pathway is linked to classical PHR transcription factor-based PSR and the link to other nutrients uptake/metabolism like N. Importantly, all these discoveries will be instrumental for future plant engineering to optimize RSA in low-P conditions.

**Figure.**
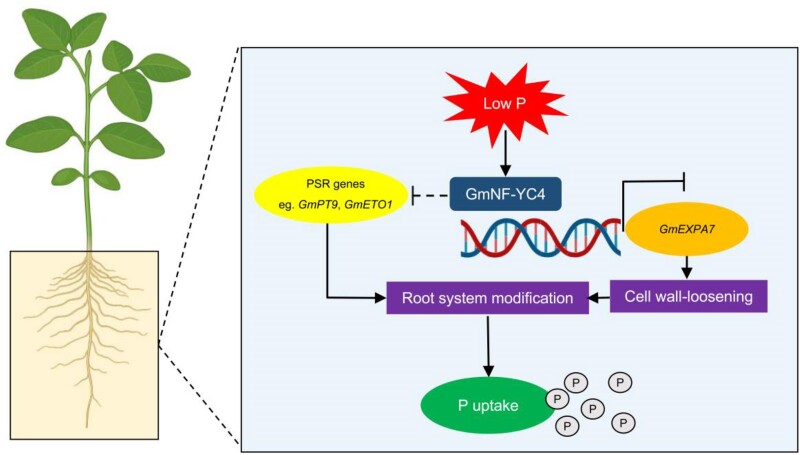
The regulatory module GmNF-YC4-GmEXPA7 controls root morphological adaptations to low-P stress in soybean. GmNF-YC4 negatively regulates root development through direct regulation of GmEXPA7 and indirect regulation of PSR genes. LP represents low-P supply (5 *μ*M P). (Figure from article being highlighted, Liu et al. (2024), Fig. 7).

## Data Availability

No new data were generated or analysed in support of this article.
